# *Notes from the Field:* Carbapenem-resistant *Klebsiella pneumoniae* with *mcr-1* Gene Identified in a Hospitalized Patient — Wyoming, January 2019

**DOI:** 10.15585/mmwr.mm6906a4

**Published:** 2020-02-14

**Authors:** Heather Rhodes, Cody Loveland, Clayton Van Houten, Noah Hull, Alexia Harrist

**Affiliations:** ^1^Epidemic Intelligence Service, CDC; ^2^Public Health Division, Wyoming Department of Health; ^3^Wyoming Public Health Laboratory.

In mid-December 2018, an adult with a history of recurrent urinary tract infections was admitted to a Wyoming hospital with acute confusion. Because of a history of methicillin-resistant *Staphylococcus aureus*, the patient was placed on contact precautions in a private room. Admission urine culture and antimicrobial susceptibility testing identified carbapenem-resistant *Klebsiella pneumoniae* with extended-spectrum beta-lactamase production. Susceptibility to colistin, an antibiotic of last resort, was not tested. Carbapenem-resistant *Enterobacteriaceae* (CRE) infections are reportable to the Wyoming Department of Health (WDH), and the isolate was sent to the Wyoming Public Health Laboratory (WPHL), where ertapenem resistance was confirmed. Further testing identified resistance to 16 antibiotics* and susceptibility to amikacin, imipenem, meropenem, and tigecycline. Using the Carba NP assay (*1*), carbapenemase production was not found. WPHL sent the isolate to the Texas Antibiotic Resistance (AR) Laboratory Network regional laboratory for further characterization. Because of known sensitivity issues with the Carba NP assay (*2*), repeat testing used the modified carbapenem inactivation method. Texas AR Laboratory Network confirmed WPHL results. Colistin susceptibility testing by broth microdilution found that the minimum inhibitory concentration was >4 *μ*g/mL, which was above the Clinical and Laboratory Standards Institute’s epidemiologic cutoff value for wild type *Enterobacteriaceae* (≤2 *μ*g/mL) (*3*). The plasmid-mediated *mcr-1* colistin resistance gene was detected using a CDC-developed multiplex real-time polymerase chain reaction assay (*4*). In early January 2019, Texas AR Laboratory Network alerted WDH that the isolate carried the *mcr-1* gene.

WDH began an investigation using CDC guidance^†^ to determine where the patient might have acquired the microorganism and to identify any spread to other hospitalized patients. WDH reviewed medical records and interviewed the patient and family members. The patient reported U.S. travel, but no international travel or livestock exposure. The patient had experienced repeated urinary tract infections with carbapenem-susceptible *Escherichia coli* and *K. pneumoniae* during the previous 2 years, which were treated with cephalosporin, fluoroquinolone, and nitrofuran antibiotics. The patient’s last hospitalization in Wyoming, for *E. coli* bacteremia, occurred 17 months earlier. In August 2018, the patient underwent a cystoscopy at a hospital-affiliated Colorado outpatient urology clinic. The Colorado Department of Public Health and Environment conducted a separate retrospective investigation into CRE reports from the clinic's geographic region; no evidence of ongoing CRE transmission was identified. The patient recovered after receiving appropriate antibiotics and was discharged in January 2019.

WDH reviewed the Wyoming hospital’s infection control measures. Medical, nursing, and infection control staff members reported adherence to contact precautions throughout the patient’s stay. A point-prevalence survey was conducted to identify possible transmission. Six patients whose hospitalizations overlapped with the index patient’s by >1 day on the same hospital unit were identified. Rectal swabs were collected from four consenting patients and sent to CDC’s Antimicrobial Resistance and Characterization Laboratory to test for *mcr-1*–positive CRE colonization. All were negative. 

The presence of *mcr-1* in CRE could promote development of untreatable, panresistant infections.^§^ Multiple countries have reported the *mcr-1* gene in *Enterobacteriaceae* species isolated from food, water, humans, and the environment (*5*), including two animal isolates and 55 human isolates in the United States.^¶^ Bacteria harboring *mcr-1* have been acquired in community and health care settings (*6*). Although *mcr-1* is less common in *K. pneumoniae* than other *Enterobacteriaceae*, presence of this transferable resistance gene in this species is of public health concern because it likely carries other antimicrobial mechanisms for resistance and is frequently associated with health care settings.**

First identified in the United States in Pennsylvania in 2016 and since then reported in 20 more states, this is the first *mcr-1* isolate reported in Wyoming or surrounding states. The patient’s frequent intermittent antibiotic use could have increased the risk for contracting antibiotic-resistant bacteria, but the route of acquisition is unknown. Wyoming’s CRE surveillance protocols supported identification of *mcr-1*. In general, laboratory testing of colistin-resistant CRE for the presence of *mcr* genes should be considered.

## Footnotes

* Resistant: trimethoprim/sulfamethoxazole, tobramycin, piperacillin/tazobactam, nitrofurantoin, levofloxacin, gentamicin, ertapenem, ciprofloxacin, ceftriaxone, ceftazidime, cefotaxime, cefepime, cefazolin, aztreonam, ampicillin/sulbactam, and ampicillin; no Clinical and Laboratory Standards Institute breakpoint: polymyxin B.

† https://www.cdc.gov/hai/containment/guidelines.html.

§ https://www.cdc.gov/drugresistance/solutions-initiative/stories/gene-reported-mcr.html.

¶ https://www.cdc.gov/drugresistance/biggest-threats/tracking/mcr.html.

** https://www.cdc.gov/hai/organisms/organisms.html#Klebsiella.
